# Short-term heavy drinking in a non-human primate model skews monocytes toward a hypo-inflammatory phenotype

**DOI:** 10.3389/fimmu.2025.1606092

**Published:** 2025-06-23

**Authors:** Madison B. Blanton, Hami Hemati, Qi Qiao, Rupak Khadka, Gregory Hawk, Kathleen A. Grant, Ilhem Messaoudi

**Affiliations:** ^1^ Pharmaceutical Sciences, College of Pharmacy, University of Kentucky, Lexington, KY, United States; ^2^ Microbiology, Immunology, and Molecular Genetics, College of Medicine, University of Kentucky, Lexington, KY, United States; ^3^ Dr. Bing Zhang Department of Statistics, College of Arts and Sciences, University of Kentucky, Lexington, KY, United States; ^4^ Division of Neuroscience, Oregon National Primate Research Center, Oregon Health and Science University, Beaverton, OR, United States

**Keywords:** monocytes, non-human primate, alcohol, hypo-inflammatory, transcriptome, epigenome

## Abstract

**Introduction:**

Alcohol use is prevalent in the United States (US), with ~80% of persons over 12 years old reporting alcohol consumption in 2023 and ~10% of those individuals developing alcohol use disorder (AUD). Acute and chronic alcohol consumption exert opposite effects on the immune system. Specifically, acute alcohol exposure (AAE), (3–16 hours of *in-vitro* treatment, one binge episode in humans, or one gavage feeding in mice) skews monocytes towards a hypo-inflammatory phenotype associated with reduced TNFα, IL-6, and MCP-1 production. In contrast, chronic alcohol consumption (CAC) (7 days of *in-vitro* treatment, 3–12 months of consumption in animal models, or humans with confirmed AUD diagnosis), shifts the functional, transcriptional, metabolic, and epigenetic landscapes of monocytes and their progenitors towards a hyper-inflammatory profile. Despite the extensive work investigating AAE and CAC, few studies have examined short-term drinking durations. We sought to bridge this gap by assessing monocytes after 6 months of ethanol consumption in a rhesus macaque model, which we considered short-term drinking. Understanding the longitudinal changes in monocytes’ phenotype and function in the context of alcohol consumption could pave the way to identifying diagnostic biomarkers for disease progression.

**Methods:**

To bridge this gap, we obtained peripheral blood mononucleated cells (PBMC) isolated from rhesus macaques before and after 6 months of daily ethanol consumption (>55% of intakes over 2.0 g/kg/day). Monocytes were analyzed using a combination of flow cytometry, single-cell RNA-sequencing (scRNAseq), ELISAs, and Cleavage Under Targets and Tagmentation (CUT&Tag).

**Results:**

Our data show that 6 months of ethanol consumption rewires monocytes towards a hypo-inflammatory profile as evidenced by reduced cytokine production. scRNAseq analysis revealed distinct shifts in monocyte states/clusters with ethanol consumption and LPS stimulation in line with a shift to a hypo-inflammatory state. These changes may be driven by reduced levels of H3k4me3, a histone modification shown to be deposited at promoter regions of genes involved in inflammation and pathogen response signaling.

**Discussion:**

Overall, these data demonstrate that 6 months of daily heavy drinking attenuates inflammatory responses in monocytes via shifts in the epigenetic landscape.

## Introduction

Alcohol misuse is prominent in the United States (USA), with ~30% of persons over the age of 18 reporting excess alcohol use in the past month (1). Alcohol consumption is a leading cause of emergency department visits (2) and preventable deaths (3) in the USA. Indeed, long-term alcohol misuse is associated with several negative health outcomes (4), notably liver (5) and cardiovascular disease (6), cancer (7), and increased susceptibility to bacterial and viral pathogens (8–15). Moreover, alcohol misuse impairs wound healing (16) and increases postoperative complications (17). Collectively, these data indicate that alcohol misuse negatively impacts the immune system (18). While significant changes in the lymphoid compartment can be observed after decades of heavy alcohol misuse, or chronic alcohol consumption (CAC) (18–20), we and others have previously shown that 12 months (12mo) of CAC results in transcriptional, functional, and epigenetic changes predominantly within myeloid cells, notably monocytes and macrophages (21). The primary roles of monocytes and macrophages are to provide antimicrobial defense and maintain tissue homeostasis (22–24). These cells exhibit a high degree of plasticity, allowing them to polarize toward a pro-inflammatory phenotype to respond to pathogens or a regulatory phenotype to mediate tissue repair (22–24). Therefore, dysregulation of these cells contributes to the pathophysiology of several diseased states (25–27). Together, these reports highlight the importance of understanding the phenotype of monocytes throughout the progression of AUD.

The impact of alcohol on the myeloid compartment is duration- and dose-dependent. Acute alcohol exposure (AAE) (3–16 hours of 25mM-150mM ethanol *in-vitro* (28, 29), *in-vivo* dose of 2mL ethanol/kg body weight consumed in 30 minutes by healthy human volunteers (30), or one-gavage feeding with a 32% ethanol solution for mice (31)) induces a hypo-inflammatory phenotype in circulating monocytes. Specifically, monocytes produce lower levels of TNFα (28, 32), MCP-1 (30), IL-8 (30), IL-1β (29), and IL-6 (31), while the production of IL-10 (29, 33) and the induction of inhibitory NF*κ*B homodimers (34) are increased. On the other hand, CAC (25mM for 7 days *in-vitro (* 35) or *in-vivo* via 12mo of daily heavy drinking in a rhesus macaque (36, 37), humans who meet the NIAAA requirements for heavy drinking or have a confirmed AUD diagnosis (38, 39)) has been shown to exert the opposite effects. Specifically, monocytes after CAC are poised toward a hyper-inflammatory phenotype indicated by increased production of inflammatory mediators to LPS and impaired ability to respond to microbial challenges as evidenced by decreased phagocytosis and immune mediator production to whole pathogen stimulation (36, 40). Similar changes were observed in tissue-resident macrophages with CAC (41–45). These aberrant responses were mediated by intrinsic changes to the transcriptional and epigenetic profiles of monocytes and macrophages (36, 46), notably increased levels of H3k4me3, a histone mark associated with active promoters in splenic macrophages (41). Moreover, CAC skews the functional (37), metabolic (47), and transcriptional (37) landscapes and the differentiation trajectory (37) of hematopoietic stem and progenitor cells (HSPCs) toward a hyper-inflammatory phenotype.

Despite the extensive work defining the phenotypes induced by acute and prolonged alcohol consumption, few studies have examined the impact of short-term, weeks to months, drinking durations. Approximately 10% of individuals over the age of 12 develop an alcohol use disorder (AUD) in the United States (48). Since myeloid cells can facilitate the development of AUD (microglia) and alcohol-induced organ damage (Kupffer cells, splenic macrophages, alveolar macrophages), it is crucial to understand the longitudinal changes in phenotype and function of these cells with alcohol consumption. To bridge this knowledge gap, we leveraged access to longitudinal peripheral blood mononucleated cells (PBMC) samples from a non-human primate model of voluntary ethanol self-administration before and after 6 months (6mo) of heavy daily ethanol consumption. Our results show that after 6mo of heavy alcohol use, monocytes are poised toward a hypo-inflammatory phenotype likely mediated by alterations in the epigenome.

## Materials and methods

### Ethics approval state

All animal studies included herein were approved by the Oregon National Primate Research Center (ONPRC) Institutional Animal Care and Use Committee (IACUC). Throughout the duration of the study, every effort was made to minimize animal discomfort per the regulations stipulated by the USDA and OLAW. For these studies, animals are trained to voluntarily present their leg for blood draws, allowing samples to be obtained within 3–5 minutes. The amount of blood taken from each animal is highly regulated, as each draw is reported on the electronic medical records. The monkeys are on a standard operating procedure for voluntary alcohol self-administration with water also available concurrently with alcohol (4% w/v, diluted in water, a concentration that is not avoided by rhesus monkeys). Moreover, they are provided food in 3 meals/day and kept in a positive weight gain throughout the protocol. They are provided 1–2 hrs a day of direct social contact and 24 hrs/day with visual and auditory access to all monkeys in the housing room. The daily drinking sessions occur within the housing cage and begin at the same time for all monkeys on protocol in the housing room.

### Animal study and sample collection

Blood samples were collected from 12 rhesus macaques (4 females, 8 males) before and after 6mo of open access to alcohol through the Monkey Alcohol Tissue Research Resource (MATRR; www.matrr.com; cohorts 7a, 16, and 18) (49). In this model, rhesus macaques (4–6 years old) are trained to use operant drinking panels and to present their legs in their home cages for blood collection. Animals go through an induction phase of 4 mo of schedule-induced polydipsia protocol with 16hr of drinking sessions followed by an “open access” phase of 6 mo with 22h/day of voluntary drinking session (4% ethanol w/v solution or water choice) as described in detail (50) and the drinking category of ethanol consumption is determined as previously described (51). Animals are individually housed in quadrant cages with constant room temperature (20-22°C), humidity (65%) and an 11hr light cycle. Cohort demographics with drinking categorization are outlined in more detail in **Table 1**.

**Table 1:** Demographics of samples used for experiments.

MATRR ID	Cohort on MATRR	Sex	Phenotype at necropsy	Average alcohol intake for the first 6mo of open access (g/kg)
10257	16	Female	VHD	2.87
10340	18	Female	VHD	3.53
10333	18	Female	HD	3.04
10256	16	Female	BD	2.07
10265	16	Male	VHD	3.94
10337	18	Male	VHD	4.02
10338	18	Male	VHD	5.93
10342	18	Male	VHD	4.04
10091	7a	Male	VHD	2.58
10098	7a	Male	VHD	3.34
10088	7a	Male	HD	2.23
10097	7a	Male	HD	2.76

Cohort demographics. BD, Binge drinking (EtOH intake ≥2 g/kg for ≥55% of open access days AND BEC ≥ 80 mg/dl at least once per year); HD, Heavy drinking (EtOH intake ≥ 3 g/kg for ≥ 20% of open access days); VHD, Very heavy drinking (daily EtOH intake >3 g/kg AND ≥ 10% of open access days with EtOH intake >4 g/kg).

### Flow cytometry

PBMC were stained with: CD3-BV510 (Clone OKT3), CD20-BV510 (Biolegend: Clone 2H7), CD14-AF700 (Clone M5E2, Biolegend), CD16-PB (Clone 3G8, Biolegend), HLADR-APC-Cy7 (Clone L243, Biolegend), CX3CR1-PE (Clone 2A9-1, Biolegend), CD86-BV605 (Clone IT2.2, Biolegend), CD163-PerCP-Cy5.5 (Clone GHI/61, Biolegend), CD169-Pe-Dazzle 594 (Clone. 7-239, Biolegend), TLR4-APC (Clone HTA125, Biolegend), and True-Stain Monocyte Block (Biolegend) for 30 min at 4°C. Samples were acquired on the Attune NxT Cytometer (ThermoFisher Scientific) and were analyzed using FlowJo (BD).

### Stimulation and intracellular cytokine staining

PBMCs were stimulated overnight (~16 hours) with a bacterial cocktail of 1ug/mL Pam3CSK4 (TLR1/2 ligand, InvivoGen), 0.5ug/mL of FSL-1 (TLR2/6 ligand, InvivoGen), and 0.5ug/mL of LPS (TLR4 ligand, InvivoGen) in the presence of Brefeldin-A (Biolegend). The following day, the cells were surface stained with CD3-FITC (Clone Sp34, Biolegend), CD20-FITC (Clone 2H7, Biolegend), CD14-AF700 (Clone M5E2, Biolegend), CD16-PB (Clone 3G8, Biolegend), HLADR-APC-Cy7 (Clone L243, Biolegend), and True-Stain Monocyte Block (Biolegend). They were then fixed, permeabilized, and antibodies against TNFα-APC (Clone Mab11, Biolegend) and IL-6-PE (Clone MQ2-6A3, BD) were added. Data were acquired on the Attune NxT Cytometer (ThermoFisher Scientific) and analysis was completed using FlowJo (BD).

### Phagocytic capacity

5x10^5^ PBMC were incubated with pHrodo Red *E. Coli* BioParticles (Thermo Fisher Scientific) for 2 hours. Cells were then stained with Ghost Dye Violet 510 (Tonbo) for 30 minutes to determine viability and then surfaced stained with CD3-FITC (Clone Sp34, BD), CD20-FITC (Clone 2H7, Biolegend), CD14-AF700 (Clone M5E2, Biolegend), CD16-PB (Clone 3G8, Biolegend), HLADR-APC-Cy7 (Clone L243, Biolegend), and True-Stain Monocyte Block (Biolegend), acquired on the Attune NxT Cytometer (ThermoFisher Scientific) and analyzed using FlowJo (BD).

### Single-cell RNA-sequencing library preparation

Monocytes were isolated using the EasySep™ APC Positive Selection Kit II (Stemcell) following staining of PBMCs with CD14-APC (Clone M5E2, Biolegend). CD14+ cells were pooled based on sampling timepoint and then incubated with or without LPS at 37°C with 5% CO2 for 4 hours. Cells were then loaded into a Chromium Controller (10x Genomics) at a concentration of 1,600 cells/uL; with the final target recovery of 30,000 cells. Library preparation was completed using the v3.1 Chromium Single Cell 3′ Kit (10x Genomics) following the manufacturer’s instructions. Libraries were sequenced at Novogene on the NovaSeq X Plus with a target of 20,000 reads per cell.

### Single-cell RNA-sequencing analysis

Samples were aligned to the rhesus macaque genome (Mmul_10) using the Cell Ranger Software (version 7.2) and downstream analysis was carried out in R 4.1.1 using Seurat (version 5.1). Aligned reads underwent initial quality control analysis by removing droplets with ambient RNA (cells with <400 detected genes), doublets (cells with >4000 detected genes) and dying cells (>5% total mitochondrial gene expression). All samples were integrated using Seurat and then normalized using NormalizeData.

The FindNeighbors and FindClusters (resolution = 0.3) function in Seurat were used to cluster the cells using the first 10 principal components and visualized via uniform manifold approximation and projection (UMAP). Clusters were identified using canonical markers from the *FindAllMarkers* functions (**Supplementary Table 1**) and contaminating B and T cells were removed for downstream analysis.

Differentially expressed genes were determined via *DESeq2* under default settings in *Seurat* and enriched using Metascape. Only statistically significant genes (average log(fold-change) cutoff >0.58 or <-0.58; adjusted p-value ≤ 0.05) were included in downstream analysis (**Supplementary Table 2**).

Gene scoring analysis was conducted by Seurat’s *AddModuleScore* function, leveraging pathways and gene signatures lists from KEGG (www.genome.jp/kegg/pathway.html) or Ensembl BioMart. Specific gene expression was determined using the AggregateExpression function. For both analysis, cells from their respective sampling timepoint/stimulation condition were randomly assigned to three groups, and the average module or expression of the group was plotted.

Slingshot was used to reconstruct monocyte trajectories across pseudotime (52) after the UMAP underwent dimensional reduction in Seurat. Cluster Mono0 was set as the root state for all downstream analysis as it is the most abundant cluster in the baseline/non-stimulated sample. DEG between groups along their respective lineages were determined using the condiments workflow adopted from (53).

### Cleavage under targets and tagmentation

Monocytes were isolated from freshly thawed PBMC using EasySep™ APC Positive Selection Kit II (Stemcell). 1.6x10^5^-2x10^5^ monocytes were used for chromatin immunoprecipitation sequencing via the CUT&Tag approach. Sample preparation for H3k4me3 (Cat # 39016, Active Motif) was constructed using Active Motif’s CUT&Tag-IT™ Assay Kit (Cat # 53160, Active Motif) following manufacturer’s instructions. Libraries were sequenced by Novogene on the NovaSeq X Plus at a depth of 10 million reads/sample.

### CUT&Tag sequencing analysis

Paired-end reads were quality-checked using FASTQC, trimmed using Trimmomatic, and aligned to the Macaca mulatta genome (Mmul10) using Bowtie2. Peak calling was carried out using MACS2. The peaks were merged using bedtools across biological replicates within timepoints (baseline and 6mo) to enhance statistical power for differential peak analysis. Due to the lack of available annotation databases for rhesus macaques, a liftover of the Rhesus macaque genome (mmul10) to the human genome (hg38) was completed using LiftOver. EpiCompare was used for the comparisons between genome-wide histone modification profiles. All peaks overlapped with hg38 blacklisted regions were removed before downstream analysis with the ENCODE identifier ENCFF356LFX. The peak overlaps heatmap and TSS plots were generated using the ENCODE identifier ENCFF133YDZ as the reference. Differential Peak analysis was completed using the HOMER’s *getDifferentialPeaks* function with a cutoff of Poisson-based p-value < 0.0001. All peaks were annotated using ChIPseeker (54) in R 4.1.1. Differentially abundant peaks at baseline and 6mo (**Supplementary Table 3**) were enriched using Metascape and Gene Set Enrichment Analysis (GSEA).

### Histone enzyme-linked immunosorbent assay

Monocytes were isolated from freshly thawed PBMC using positive selection magnetic activated cell sorting (Miltenyi Biotec). Histones were extracted from isolated monocytes using Abcam’s Histone Extraction kit (Abcam) per manufacturer’s instructions and final quantification of protein concentration was assessed using the Qubit Protein Assay Kit. The level of total H3 histone and respective histone modifications were measured with Abcam’s Histone H3 Modification Multiplex Assay Kit (colorimetric) (Abcam). Only select modifications were tested (H3K4me2, H3K4me3, H3k9me1, H3k9me2, H3K9me3, H3K27me1, H3K27me2, H3K27me3), utilizing 500ng of protein from each sample per reaction. Data were acquired using a SpectraMax iD3 multi-mode plate reader and final calculations using OD values were completed as outlined per the manufacturer.

### Statistical analysis

All statistical comparisons in this manuscript were completed in R 4.3.1 unless otherwise stated in the methods. Differences between timepoints (baseline vs. 6mo of CAC) or response to challenge (non-stimulated vs. stimulated) were determined using a linear mixed-model approach. All model assumptions, including normality, were assessed using a combination of visual plots, including residual plots and histograms. Differences in cell cluster frequencies in the scRNA-seq data set were determined using a one-way ANOVA with unmatched samples. The Šídák test was used to correct for multiple comparisons. Values for module scores were tested using an unpaired t-test with Welch’s correction. All bar plots depict the mean **±** SEM. A p-value of <0.05 was considered significant.

## Results

### 6mo of heavy drinking skews circulating monocytes toward a hypo-inflammatory phenotype

**Figure 1:**
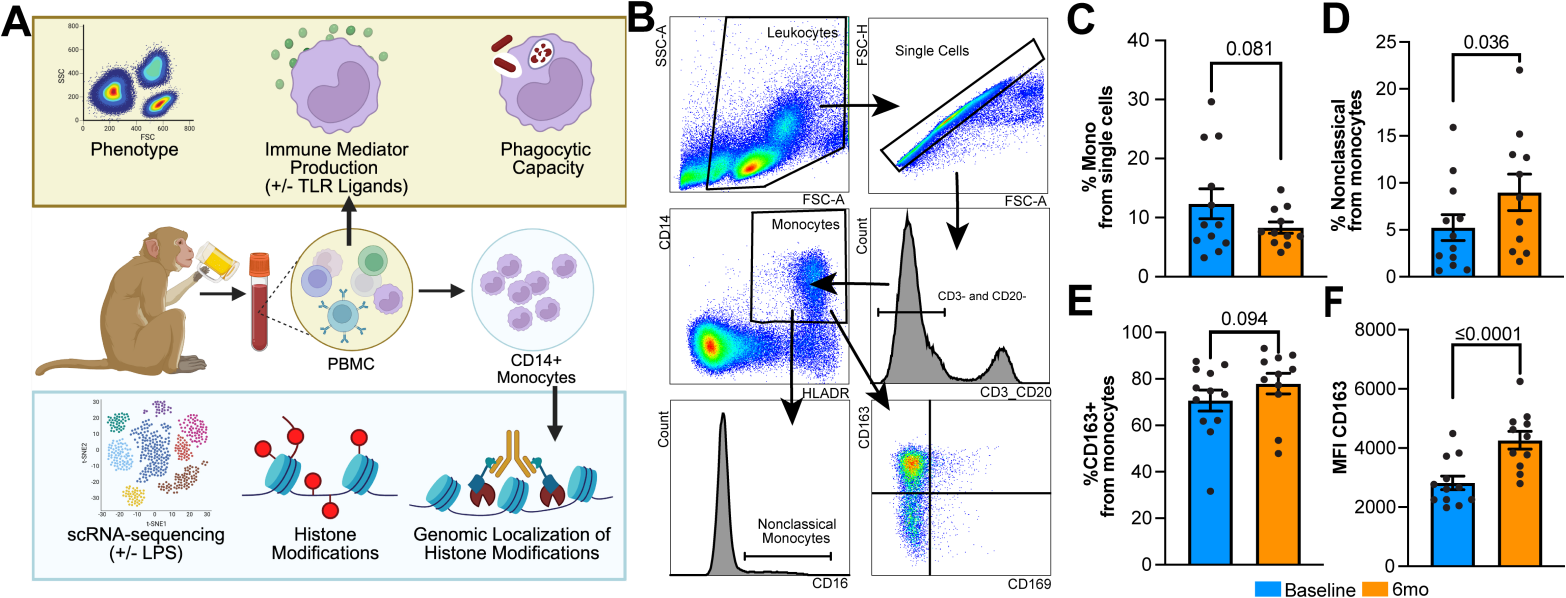
Circulating monocytes display a hypo-inflammatory phenotype after 6mo of heavy ethanol consumption. **(A)** Study design. **(B)** Representative gating strategy used to identify and phenotype monocytes within peripheral blood mononuclear cells (PBMC). Bar plots indicating the **(C)** change in the overall percentage of monocytes, **(D)** percentage of nonclassical monocytes within the monocyte population, and **(E)** percentage of CD163+ monocytes and **(F)** their respective MFI. Statistical significance was determined using a linear mixed model, and error bars were defined as ± standard error of the mean (SEM). A p-value of <0.05 was considered significant while a value 0.1>x>0.5 was denoted as trending.

To understand how short-term durations of heavy alcohol use affect the profile of circulating monocytes, PBMC obtained before and after 6mo of daily, heavy ethanol consumption were phenotyped using key canonical surface markers (**Figures 1A, B**). After 6mo of drinking, there was a modest decrease in the frequency of circulating monocytes after alcohol use compared to baseline (**Figure 1C**) that was accompanied by a significant increase in the proportion of nonclassical monocytes. (**Figure 1D**). While the frequency of CD163+ monocytes, representing a hypo-inflammatory state, was not significantly changed after 6mo of daily alcohol use, the expression level of CD163 was significantly enhanced by alcohol use, indicated by increased mean fluorescent intensity (MFI) (**Figures 1E, F**). Additionally, no differences were noted in the frequency of cells expressing CD169, CX3CR1, CD86, or TLR4 or MFI of expression (data not shown).

**Figure 2:**
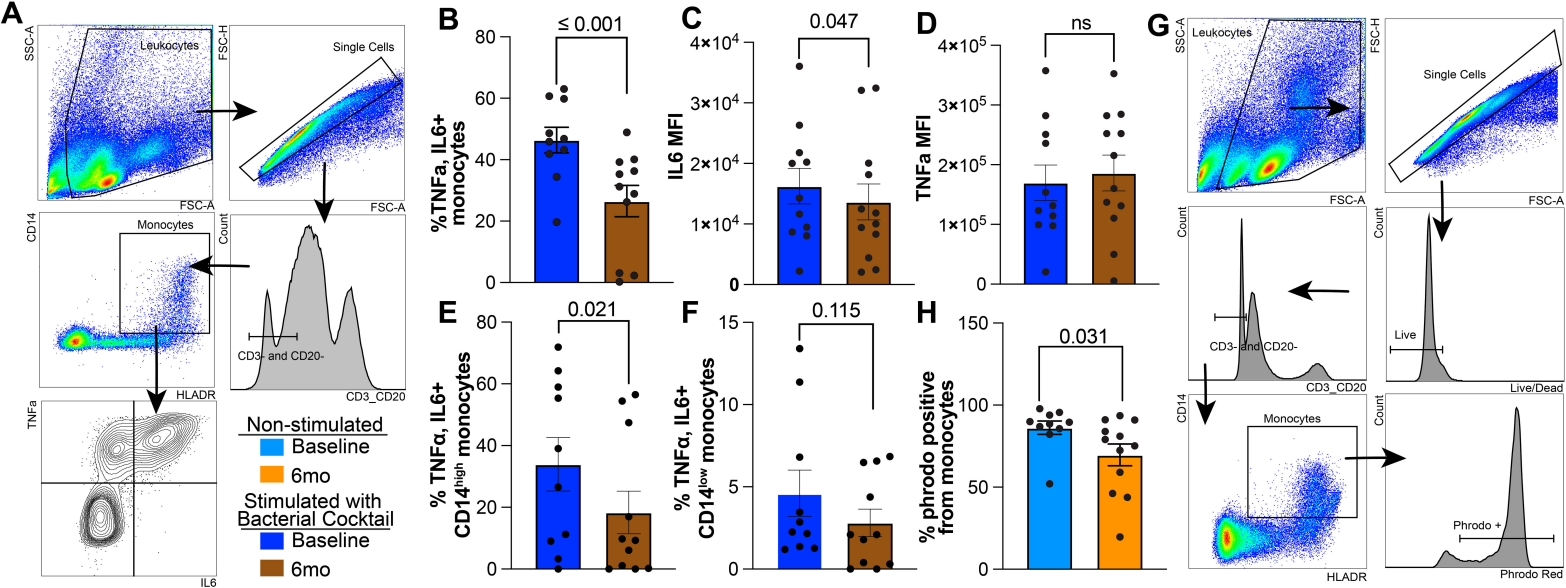
Reduced IL6 production and phagocytosis in circulating monocytes after 6 months of chronic ethanol consumption. **(A)** Gating strategy used to identify TNFα+ and IL6+ monocytes. **(B)** Bar blots comparing the corrected percentage of TNFα+IL6+ monocytes before and after 6mo of heavy ethanol consumption. MFI of **(C)** IL6 and **(D)** TNFα from TNFα+IL6+ monocytes after stimulation with bacterial ligands. Bar plot representing the percentage of TNFα+IL+ **(E)** CD14^high^ and **(F)** CD14^low^ monocytes. **(G)** Representative gating strategy used to identify Phrodo Red positive (phrodo+) monocytes. **(H)** Bar plots indicating the percentage of phrodo+ monocytes. Statistical significance was determined using a linear mixed model, and error bars were defined as ± standard error of the mean (SEM).

Next, we challenged the cells with a cocktail of bacterial ligands (Pam3CSK4, FSL-1, and LPS) to emulate the complexity of bacterial pathogens and measured production of IL-6 and TNFα (**Figure 2A**). Following stimulation, a lower percentage of monocytes produced TNFα and IL-6 after 6mo of heavy drinking compared to baseline (**Figure 2B**). Moreover, levels of IL-6, but not TNFα, produced by monocytes were also reduced after 6mo of drinking compared to baseline (**Figures 2C, D**). This alteration in immune mediator production stemmed from a significant reduction TNFα and IL6 production by CD14^high^ monocytes, as there were no changes observed in the CD14^low^ subset (**Figures 2E, F**, **Supplementary Figure 1A**). Additionally, the overall ability of monocytes to phagocytose *E. coli* conjugated bioparticles was decreased after short-term alcohol consumption (**Figures 2G, H**). Both CD14^high^ and CD14^low^ subsets contributed to this reduced functional output (**Supplementary Figures 1A, B**). Taken together, these data suggest that 6mo of heavy drinking results in increased generation of hypo-inflammatory monocytes, reduced production of inflammatory mediators, and attenuated phagocytic potential.

### 6mo of heavy drinking induce a unique transcriptional state in monocytes

**Figure 3:**
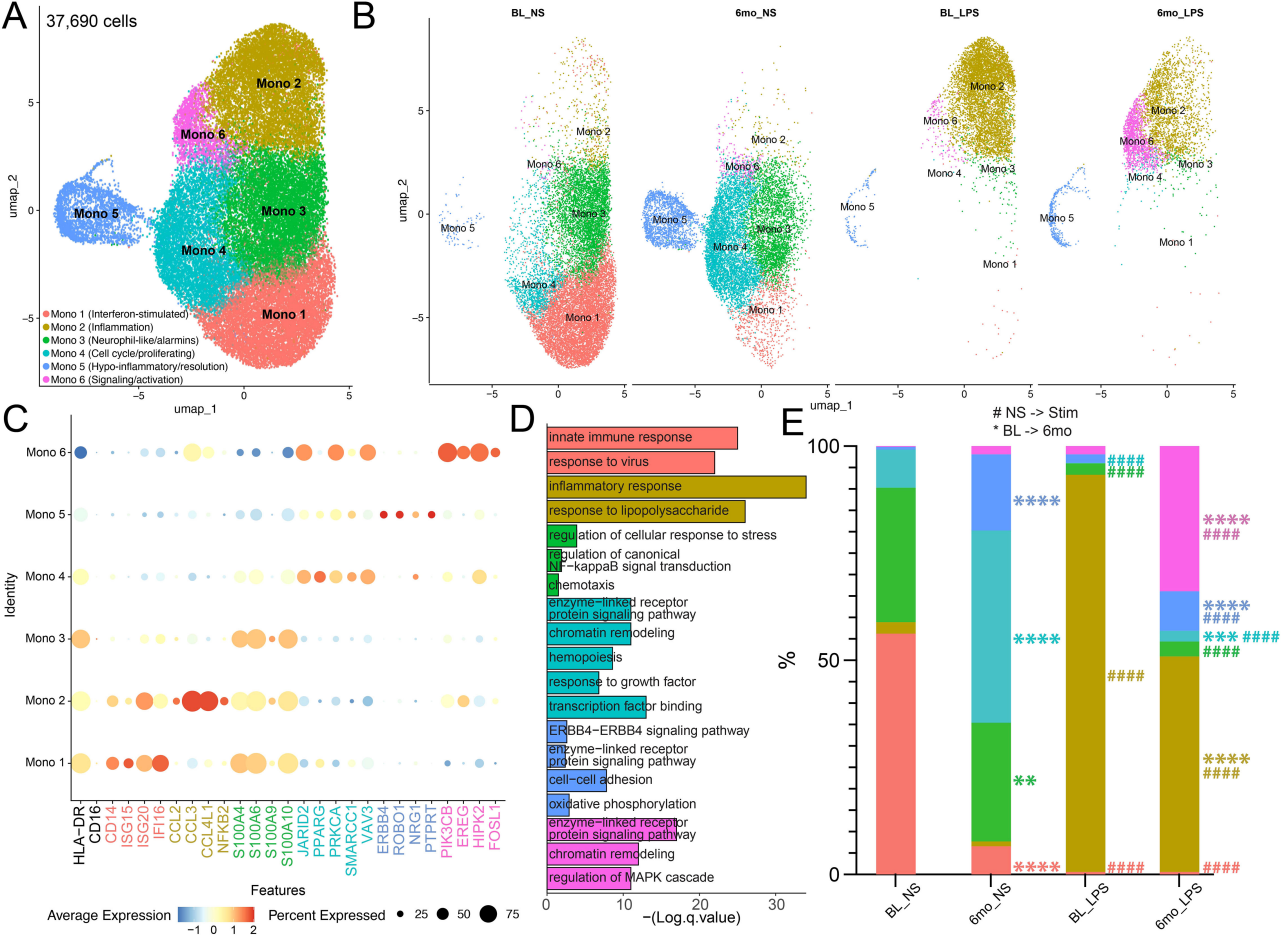
Heavy ethanol consumption for 6 months and LPS stimulation drive distinct monocyte sub-populations. **(A)** Uniform Manifold Approximation and Projection (UMAP) of 37,690 CD14+ monocytes. **(B)** UMAPS displaying the contribution of cells from respective time points/stimulation conditions for each cluster. **(C)** Selected marker genes used to identify clusters. **(D)** Functional enrichment of all marker genes by cluster. **(E)** Relative abundance of clusters stratified by timepoint/stimulation condition (* indicates differences between BL and 6mo; # represents differences between NS and LPS stimulation). A one-way ANOVA between unmatched samples with Šídák correction for multiple comparisons was used to determine differences in cell cluster frequencies across each sample. All bar plots depict the mean ± SEM. A p-value of <0.05 was considered significant. ** p-value <0.0021; *** p-value <0.0002; **** p-value <0.0001; #### p-value <0.0001.

Next, we sought to understand the molecular mechanisms driving phenotypic and functional changes after 6mo of heavy drinking by performing single-cell RNA sequencing (scRNAseq) on isolated monocytes before and after 6mo of ethanol consumption in the presence and absence of LPS stimulation, a well-studied and effective activator commonly used to assess monocyte function following alcohol exposure. Uniform manifold approximation and projection (UMAP) revealed six distinct clusters with varying frequencies across timepoint and stimulation conditions (**Figures 3A, B**). All clusters expressed *HLADR*, with minimal or no *FCGR3* (*CD16*) expression, suggesting they are predominantly classical monocyte clusters (**Figure 3C**). Interestingly, we were unable to detect a cluster with high expression of *FCGR3* (CD16) in this data set. Cluster Mono 1 was characterized by high expression of interferon-stimulated genes (ISG; *ISG15, ISG20, IFI16)*, whereas Mono 2 expressed high levels of chemokines (*CCL2, CCL3, CCL4L1, NFKB2)*. Mono 3 marker genes included predominantly alarmins (*S100A4, S100A6, S100A9, S100A10)*, while marker genes of Mono 4 play critical roles in cell-cycle (*JARID2, PPARG, PRKCA, SMARCC1, VAV3)*. Marker genes of Mono 5 are involved in the resolution of inflammation (*ERBB4, ROBO1, NRG1, PTRRT)*. Finally, cells in Mono 6 expressed high levels of genes that are involved in key signaling pathways/cell activation (*PIK3CB, EREG, HIPK2, FOSL1)* (**Figure 3C**). Functional enrichments of marker genes specific to each cluster were used to gain insight into the functions of the clusters. In line with the high expression of ISG, genes defining Mono 1 enriched to gene ontology (GO) terms associated with anti-viral immune responses (**Figure 3D**). Marker genes of Mono 2, Mono 3, and Mono 6 mapped to pathways involved in anti-bacterial response and inflammatory signaling (**Figure 3D**). Marker genes of Mono 4 mapped to cell cycling while those of Mono 5 enriched to hypo-inflammatory feedback mechanisms (**Figure 3D**).

**Figure 4:**
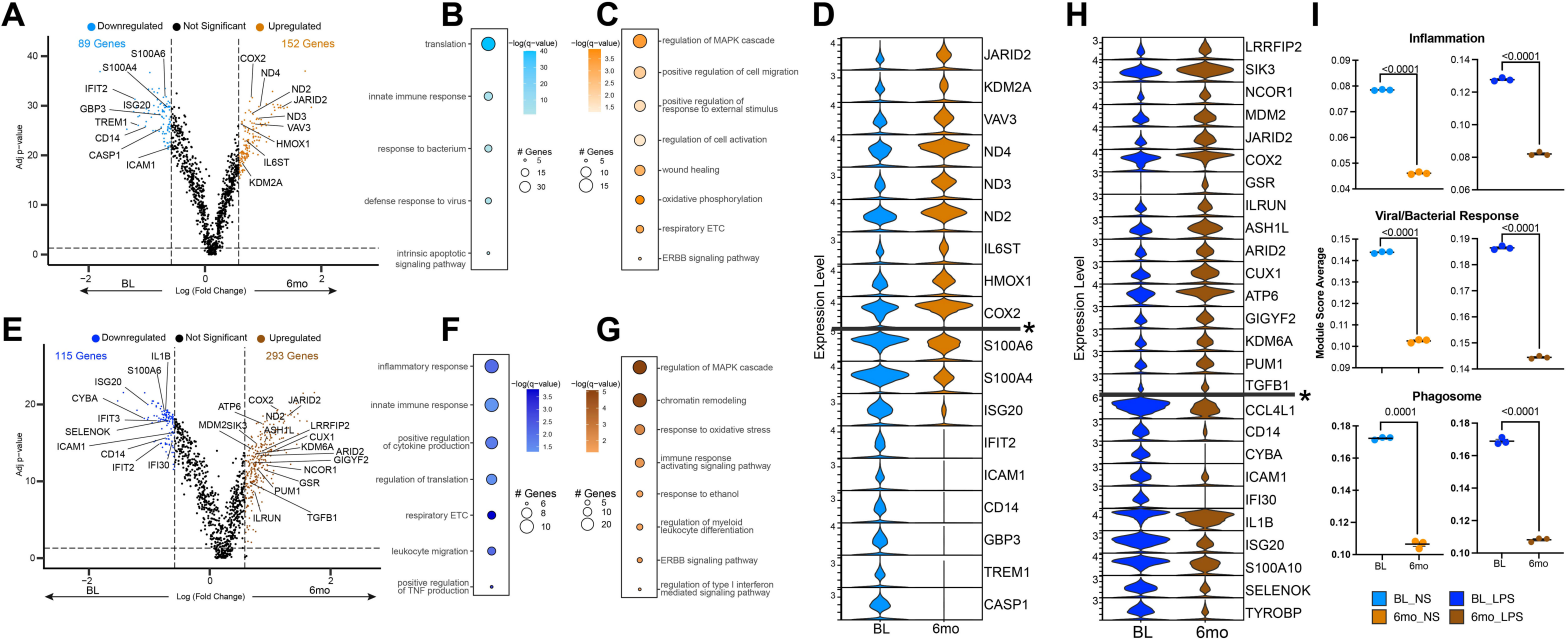
Altered monocyte transcriptional profiles after 6 months of chronic ethanol consumption. **(A)** Volcano plot depicting genes differentially expressed gene (DEG) in non-stimulated monocytes after 6mo of drinking. **(B, C)** Functional enrichment of DEG in non-stimulated monocytes at baseline **(B)** and after 6mo of heavy ethanol use **(C)**. **(D)** Violin plot of selected DEG from non-stimulated monocytes. Genes below the * are significantly more expressed in the baseline samples, and genes above are significantly more expressed after 6mo of heavy drinking. **(E)** Volcano plot depicting DEG in LPS stimulated monocytes after 6mo of drinking. **(F, G)** Functional enrichment of DEG in LPS-stimulated monocytes at baseline **(F)** and after 6mo of heavy ethanol use **(G)**. **(H)** Violin plot of selected DEG from LPS stimulated monocytes. Genes below the * are significantly more expressed in the baseline samples, and genes above are significantly more expressed after 6mo of heavy drinking. **(I)** Functional module scores between sampling time points at rest (left) and with LPS stimulation (right). Differentially expressed genes were determined via *DESeq2* under default settings in *Seurat* and enriched using Metascape. Only statistically significant genes (average log(fold-change) cutoff >0.58 or <-0.58; adjusted p-value ≤ 0.05) were included in downstream analysis. Values for module scores were determined using an unpaired t-test with Welch’s correction. All bar plots depict the mean ± SEM. A p-value of <0.05 was considered significant.

Non-stimulated monocytes at baseline (BL) were primarily composed of Mono1 and Mono 3 clusters (**Figures 3B, E**). However, 6mo of heavy drinking resulted in a significant decrease in Mono 1 and 3 while the frequency of Mono 4 (proliferating) and 5 (regulatory phenotype) increased (**Figures 3B, E**). To uncover the impact of 6mo of heavy drinking on monocyte transcriptome, we collapsed all clusters within their respective stimulation condition and sampling timepoint to ensure robust differential gene expression analysis (**Supplementary Table 2**). First, to understand how 6mo of daily heavy drinking rewires the transcriptional landscape of monocytes, we compared the unstimulated transcriptional landscape of monocytes at baseline and after 6mo of drinking (BL_NS vs. 6mo_NS). We observed 241 differentially expressed genes (DEG) (**Figure 4A**), suggesting 6mo of ethanol consumption is sufficient to induce heterogeneity within the monocyte transcriptome. There were 89 genes with higher expression in BL samples, enriching to gene ontology (GO) terms associated with pathogen response pathways (ex. *TREM1, ISG20, CD14, GBP3*) (**Figures 4A, B, D**). In contrast, after ethanol consumption, we see an increase in the expression of 152 genes involved with inflammation regulation/resolution (ex. *JARID2, KDM2A*) and homeostatic metabolic pathways such as oxidative phosphorylation (ex. *ND2, ND3, ND4*) (**Figures 4A, C, D**).

### 6mo of heavy drinking rewires monocyte transcriptional response to LPS

Following LPS stimulation of BL samples, the frequency of the ISG (Mono 1), neutrophil-like (Mono 3), and proliferating (Mono 4) clusters decreased with a concomitant increase in inflammatory monocytes (Mono 2) (**Figures 3B, E**). Similarly, LPS stimulation of samples after 6mo of alcohol consumption, led to the increase of inflammatory monocytes (Mono 2) as well as Mono 6 which expressed high levels of signaling/activation genes. When comparing the frequency of clusters at BL and 6-months following LPS (BL_LPS vs 6mo_LPS), we observed a higher frequency of regulatory (Mono 4), resolution (Mono 5), and signaling/activation (Mono 6) clusters, while that of the inflammatory (Mono 2) subset was lower in the ethanol group (**Figures 3B, E**).

We then assessed the post-LPS transcriptional differences before and after 6mo of drinking (BL_LPS vs. 6mo _LPS) (**Figure 4E**). The 115 genes upregulated in the BL samples enriched to key innate immune cell processes, including cytokine production (ex. *IL1B, TYROBP*) and migration (ex. *ICAM1, CCL4L1*) (**Figures 4E, F, H**). These changes in gene expression align with our functional data showing increased functional response to LPS at BL (**Figure 2**). The 293 genes upregulated in the 6mo samples played a role in epigenetic regulation (ex. *KDM6A, ARID2*), resolution of inflammation (ex. *TGFB1, ASH1L, ILRUN*) (**Figures 4E, G, H**) as well as immune signaling (ex. *LRRFIP2, MDM2, COX2*) (**Figures 4E, G, H**) in agreement with the dampened IL-6 production. In line with the differential gene expression, scores of gene modules associated with inflammation, viral/bacterial, and phagosome responses were decreased at 6mo, regardless of stimulation condition (**Figure 4I**).

Next, we compared the transcriptional response to LPS by evaluating changes in gene expression after stimulation respective to their non-stimulated control (BL_NS vs. BL_LPS) (6mo_NS vs. 6mo_LPS) and then comparing genes that were differently expressed (**Supplementary Figure 2**). We identified 236 DEG in response to LPS at BL and 170 DEG following LPS stimulation after 6mo of heavy drinking, with the majority of the DEG shared between the two sampling time points (**Supplementary Figure 2A**). Shared DEG enriched to gene ontology (GO) terms associated with immune responses (ex. *IL1B, CXCL8, CCL3, TGFB1)* and signaling (ex. *IRAK2, C5AR1, PIK3AP1*) (**Supplementary Figure 2B**). DEG unique to BL enriched to pathways involved in IL-6 production (ex. *SELENOK, RABGEF1, IL6R)* and regulation of gene transcription (ex. *EP300*) (**Supplementary Figures 2C, D**). On the other hand, DEG exclusive to 6mo time point are involved in negative regulation of inflammatory signaling (ex. *NFBK1, LYN*) (**Supplementary Figure 2E**). These data strongly suggest that 6mo of ethanol consumption induces a hypoinflammatory state in circulating monocytes.

**Figure 5:**
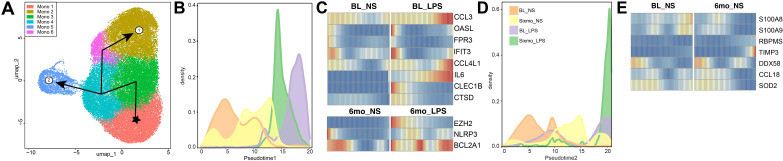
Ethanol consumption and LPS stimulation drive monocyte differentiation along different trajectories. **(A)** UMAP indicating the two distinct lineage trajectories identified using Slingshot. **(B, D)** Progression graph showing the density of samples across pseudo time and the **(C, E)** differential gene expression (DEG) between conditions along its trajectory, respective to Lineage 1 **(B, C)** and Lineage 2 **(D, E)**.

We then performed a trajectory analysis to gain insight into which genes drive the differentiation of monocytes in response to alcohol consumption and LPS stimulation. The six monocyte clusters were ordered by pseudotime, originating in Mono 1. This cluster was used to root the analysis as Mono 1 had the highest cluster frequency in the baseline and non-stimulated sample. We identified two trajectories leading to Mono 2 or Mono 5 as their terminal points (**Figure 5A**, **Supplementary Figures 3A, B**). Lineage 1, ending in Mono 2, is driven by LPS stimulation, evidenced by the high contribution of LPS-stimulated cells at the end of the pseudotime (**Figure 5B**). DEG between non-stimulated and LPS-challenged groups across pseudotime indicates that this trajectory is driven by an increase in the expression of genes involved in antimicrobial responses including chemokines and components of the inflammasome (*CCL3, CCL4L1, IL6, NLRP3)* (**Figure 5C**). Lineage 2 culminates in Mono 5, a cluster with a high density of cells after 6mo of heavy alcohol use (**Figures 3B, E**, **5D**). Differential gene expression for lineage 2 indicates a significant reduction in the expression of inflammatory genes (*S100A8, S100A9, SOD2*) across pseudotime (**Figure 5E**). These data show that 6mo of heavy drinking skew the transcriptome of monocytes towards a hypo-inflammatory state.

### Alcohol-induced histone modifications could drive alterations in functional and transcriptional profiles

**Figure 6:**
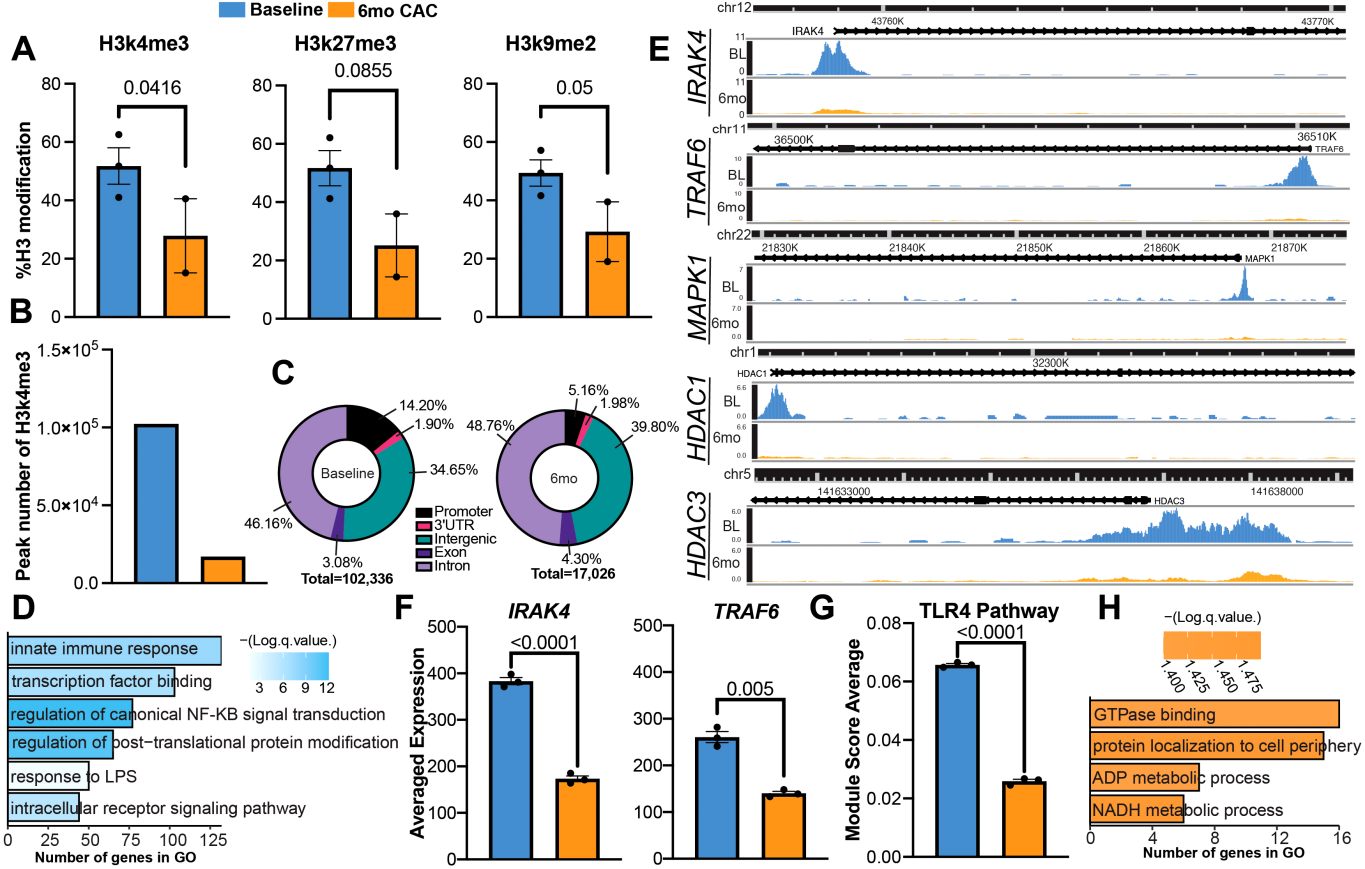
Heavy ethanol consumption for 6 months induces histone modification in circulating monocytes. **(A)** Bar plots representing the expression of histone modification determined via ELISA before and after 6mo of ethanol. **(B)** Number of differentially abundant peaks identified with H3k4me3 before and after 6mo of heavy drinking. **(C)** Genomic distribution of peaks. **(D, E)** Enrichment of peaks more abundant in the promoter region at baseline and **(E)** example pileups of respective genes. **(F)** Average expression of genes from scRNAseq. **(G)** Bar graph of TLR4 signaling pathway module score. **(H)** Enrichment of peaks more abundant in the promoter region at 6mo. Statistical significance was determined for histone modifications using a linear mixed model. Differential Peak analysis for CUT&Tag was completed using the HOMER’s *getDifferentialPeaks* function with a cutoff of Poisson-based p-value < 0.0001. Values for module scores were determined using an unpaired t-test with Welch’s correction. All bar plots depict the mean ± SEM. A p-value of <0.05 was considered significant.

To further understand the molecular underpinnings of the transcriptional changes after 6mo of heavy drinking, we investigated the expression of post-translational histone modifications via a multiplex ELISA kit. As lysine modifications were predominantly reported to regulate monocyte function (55–57), we focused on lysine residues previously shown to be modulated with alcohol use (58, 59). The abundance of activation histone mark H3k4me3 and repressive histone marks H3k9me2 were decreased at 6mo, along with a modest decrease in the deposition of repressive mark H3k27me3 (**Figure 6A**).

To identify the genes impacted by the changes in H3k4me3 deposition, we performed the Cleavage Under Targets & Tagmentation (CUT&Tag) assay. As CUT&Tag is a newer technique used to evaluate DNA-protein interactions, we first benchmarked our data using EpiCompare against a published H3k4me3 profile of healthy monocytes from ENCODE (**Supplementary Figure 3**). Overall, our peak recovery and feature distribution are comparable to the ENCODE reference and supporting literature (60). Although there is limited overlap between the peaks identified in the monocyte reference and the 6mo timepoint, a similar trend is also observed when comparing the baseline and 6mo timepoints. This suggests the discrepancy in peak calling is likely a result of ethanol consumption. When evaluating the H3k4me3 signature at the genomic level, we observed more differentially abundant genes at baseline compared to after 6mo of alcohol consumption (**Figure 6B**). Moreover, the deposition of H3k4me3 in the promoter region was decreased after alcohol use while the deposition in intergenic regions, exons, and introns increased (**Figure 6C**). We identified 5,870 peaks that were more abundant in the promoter region at baseline and 482 peaks that were abundant in the same region after 6mo of heavy drinking (**Supplementary Table 3**). Peaks that were more abundant at baseline regulated genes important to innate immune responses and epigenetic remodeling such as *IRAK4*, *TRAF6*, *MAPK1*, and *HDAC1* (**Figures 6D, E**). IRAK4 and TRAF6 are key components of the signaling cascade initiated by LPS binding to TLR4. Therefore, we compared their expression between baseline and after 6 months of ethanol access using the scRNA-seq data. A decrease in *IRAK4* and *TRAF6* transcript counts were detected after 6mo of ethanol consumption (**Figure 6F**). Moreover, TLR4 signaling module score was also reduced after 6 months of ethanol consumption indicating decreased expression of TLR4 signaling cascade components (**Figure 6G**, **Supplementary Table 4**). In contrast, peaks that were more abundant after 6mo of heavy drinking overlapped genes important for key metabolic pathways such as ADP and NADH processes, which have been shown to orchestrate innate immune cells’ ability to regulate and resolve inflammation (61, 62) (**Figure 6H**).

## Discussion

Alcohol misuse disrupts the immune system, most notably monocytes and macrophages, which are key players in antimicrobial responses and tissue homeostasis (22–24, 27). Acute and chronic alcohol consumption exert opposite effects on monocytes, with acute consumption leading to a hypo-inflammatory phenotype while long-term exposure results in a hyper-inflammatory profile (18, 20). However, few studies have investigated the impact of short-term periods of alcohol consumption on monocyte function. Therefore, in this study, we examined changes in circulating monocytes’ functional, transcriptional, and epigenetic landscapes by profiling monocytes isolated from a non-human primate model of voluntary ethanol self-administration before and after 6mo of daily, heavy alcohol consumption.

While murine and rodent models have been key to understanding the interplay between alcohol and the immune system, they do present limitations. Mouse models require an ethanol dosage of 2-5x that of humans to reach comparable peak blood concentrations (63), likely resulting from differences in the rate of ethanol catabolism between species (64). Moreover, it is also critical to note that routes of administration utilized in studies with mice and rodents, including liquid-only ethanol diets and gavage feeding with high concentrations of ethanol, are not common methods of alcohol consumption in humans. While these administration methods have been instrumental in uncovering how excess alcohol consumption impacts the liver, they have several limitations, including increased stress, which can independently modulate the immune system (65). The outbred non-human primate model bridges the gap between inbred murine models and *in vitro* studies using human cells.

Data presented herein show that 6mo of heavy drinking in the non-human primate model system was associated with an increase in non-classical monocytes, a subset associated with inflammation resolution and vascular homeostasis (66, 67). These observations align with previous reports showing an expansion of non-classical monocytes following a binge drinking episode in humans (68, 69). Non-classical monocytes can extravasate into tissues and differentiate into macrophages, albeit biased toward a regulatory phenotype (70) that is more important for tissue repair (71). Indeed, our findings indicate that 6mo of alcohol consumption led to an increase in the expression of CD163, a scavenger receptor that is enhanced in a hypo-inflammatory environment (72, 73). Similar trends in CD163 expression have been observed following an *in-vivo* acute binge episode in humans (69). Although some reports suggest that CD163 expression on macrophages is enhanced with chronic inflammation (73, 74), it is likely a result of the accumulation of alternatively activated macrophages needed for resolution rather than a maker of inflammation (75). The skewing towards a hypo-inflammatory phenotype was further evidenced by decreased production of IL-6 in response to LPS and reduced phagocytic capacity. Taken together, these data suggest that 6mo of heavy, daily drinking skews monocytes toward a hypo-inflammatory phenotype, reminiscent of that observed after acute alcohol exposure (29, 30, 76, 77).

We then leveraged scRNA-seq to gain insight into the mechanisms by which 6mo of heavy drinking disrupts monocyte function. In contrast with the phenotyping data, we did not observe a distinct non-classical monocyte population, likely a consequence of our CD14 positive selection sort as non-classical monocytes have very low CD14 expression (78). Significant shifts in cell states were observed after 6mo of drinking, with the loss of an ISG subset and the enrichment of a subset expressing high levels of hypo-inflammatory and regulatory genes, including those within the epidermal growth factor family of receptor kinases (ErbBs) (79, 80), which are critical for cell migration, differentiation, and survival (81). Specifically, expression of *ERBB4* was increased in the 6mo samples. *ERBB4* knockout mice have increased colitis, likely mediated by increased macrophage activation and inflammatory mediator production (79). Other members of the ErbB family have also been shown to attenuate monocyte TNFα production upon LPS stimulation. Thus, increased expression of genes mapping to ErbB signaling could contribute to the hypo-inflammatory functional responses observed after 6mo of drinking. Upon LPS stimulation, a significantly higher frequency of monocytes expressing inflammatory genes were present at baseline compared to 6mo, supporting our functional data of reduced immune mediator production and phagocytosis after short-term drinking. LPS stimulation after 6mo of drinking instead induced a unique cluster expressing genes involved in activation and cell signaling. Thus, the data in this study suggest that after 6mo of drinking, monocytes may begin to undergo rewiring that alters their transcriptional response capacity, leading to alterations in response pathways.

Gene expression is tightly regulated by post-translational modifications such as histone modifications (82). It has been shown that alcohol and its metabolites can induce a diverse range of histone methylations and acetylations in a variety of different organs, including the brain (83), liver (84), spleen (41), and lung (85). We have previously shown that an increase in the active promoter mark H3k4me3 after 12 months CAC in splenic macrophages correlated with increased chromatin accessibility at promoters, regulating inflammatory responses (41). In contrast, in this study, H3k4me3 deposition was reduced after 6mo of drinking, suggesting a decrease in active promoters. At baseline, we see increased deposition associated with genes within the TLR signaling cascades (*IRAK4, TRAF6*) (86). Previous work has shown that IRAK4 and TRAF6 are necessary for IL-6 production after TLR4 stimulation (87–89) and that an *in vitro* dose of acute alcohol suppresses inflammation by increasing IRAK-M, a negative regulator in the TLR4 signaling cascade, while chronic use induces an opposing effect (35). Similarly, our data shows decreased *IRAK4* and *TRAF6* gene expression and module scoring of the TLR4 signaling cascade after 6mo of daily heavy drinking. We postulate that the decreased deposition of the activating mark H3K4me3 after 6mo of drinking could lead to reduced expression of key proteins in the TLR4 signaling cascade, resulting in the attenuated IL-6 production we observed after stimulation. Interestingly, we also observed a reduction in the total deposition of repressor marks H3k27me3 and H3k9me2, in line with prior studies showing decreased H3k9me2 in the amygdala and within hepatocytes after acute alcohol consumption (90).

As previously discussed, the dose and duration of alcohol consumption induce opposite changes in monocyte phenotype and inflammatory responses. Our data demonstrate this concept as, in contrast to 12mo of heavy drinking (36, 41), 6mo of heavy drinking attenuates inflammatory responses. It is well established that prolonged drinking disrupts the intestinal barrier, allowing microbial products to ‘leak’ into circulation (91–93). Indeed, we reported elevated levels of circulating LPS and IgM-bound endotoxin after 12mo of heavy drinking in this model (36, 94). Monocytes exposed to low levels of endotoxin have been shown to have increased deposition of activating histone marks and an enhanced inflammatory response following a secondary challenge (95–97).

This study is not without limitations. Although our experiments accounted for sex as a biological variable by utilizing samples from both male and female macaques, we were not statistically powered to assess if there were unique, sex-dependent phenotypes. Our phagocytosis data should be interpreted with caution, as the use of conjugated bioparticles does not fully recapitulate antimicrobial mechanisms. Therefore, functional assays measuring intracellular killing, ROS production, phagosome acidification, and antigen presentation should be used in future studies to better assess how short-term drinking influences pathogen clearance. We have previously demonstrated that the inflammatory phenotype monocytes acquire after 12mo of alcohol misuse extends to the tissue-resident macrophages (36, 41, 42). The analysis of future studies should investigate if tissue-resident macrophages after 6mo of heavy alcohol consumption are skewed towards a hypo-inflammatory phenotype. Moreover, due to the bulk nature of our CUT&Tag experiment, we cannot determine the epigenetic landscape of specific monocyte populations. Future studies should focus on single-cell genomic localization of histone modifications and chromatin accessibility to better understand the diverse epigenetic landscape within the monocyte population. It would also be interesting to evaluate changes in the metabolic profile, as our transcriptional and epigenetic data suggest the rewiring of the metabolic landscape after 6mo of alcohol misuse.

In summary, this study provides an in-depth look at monocytes’ functional, transcriptional, and epigenetic landscape after a short-term period of daily, heavy drinking. Our data supports the conclusion that 6mo of heavy drinking in non-human primates poised monocytes toward a hypo-inflammatory phenotype. These data help to characterize the stages of dysregulation in the immune system throughout the development of AUD. This knowledge, alongside future longitudinal studies in this model system, will be useful in establishing a progression of cell phenotype or potential biomarkers to track disease development.

## Data Availability

The datasets presented in this study can be found in online repositories. The names of the repository/repositories and accession number(s) can be found below: https://www.ncbi.nlm.nih.gov/, PRJNA1231088.

## Supplementary material

Supplementary Figure 1 CD14^high^ and CD14^low^ monocyte subsets equally contribute to the decrease in phagocytosis after 6mo. (A) Representative gating strategy used to determine the contribution of monocyte subsets to functional response changes. (B) Bar plots representing the percentage of Phrodo Red positive (phrodo+) CD14^high^ and CD14^low^ monocytes. Statistical significance was determined using a linear mixed model, and error bars were defined as ± standard error of the mean (SEM). A p-value of <0.05 was considered significant.

Supplementary Figure 2 Chronic ethanol consumption for 6 months drives differential gene expression in response to LPS. **(A)** Venn diagram comparing the differentially expressed genes (DEG) in response to LPS before (BL_NS vs. BL_LPS) and after 6mo of alcohol use (6mo_NS vs. 6mo_LPS). **(B)** Functional enrichments of DEG that were shared between the response to LPS at baseline and after 6mo of drinking and **(C)** exclusive to baseline response. **(D)** Select genes specific to LPS response at baseline and **(E)** after 6mo of heavy drinking. Differentially expressed genes were determined via *DESeq2* under default settings in *Seurat* and enriched using Metascape. Only statistically significant genes (average log(fold-change) cutoff >0.58 or <-0.58; adjusted p-value ≤ 0.05) were included in downstream analysis.

Supplementary Figure 3 LPS stimulation and chronic ethanol consumption induce distinct differentiation trajectories. Heatmap of top 100 genes across pseudotime driving **(A)** Lineage 1 and **(B)** Lineage 2.

Supplementary Figure 4 Quality control of the CUT&Tag analysis. **(A)** Correlation of sample peaks to ENCODE monocyte H3k4me3 peak ranges. **(B)** Genomic distribution of total peaks identified in our samples and the ENCODE monocyte H3k4me3 reference. **(C)** Peak counts at the transcription start site.

Supplementary Table 1 Cluster markers calculated using the *FindAllMarkers* function in Seurat.

Supplementary Table 2 List of differentially expressed genes.

Supplementary Table 3 Differentially abundant peaks in the promoter region from CUT&Tag.

Supplementary Table 4 Genes for TLR4 module score.
