# Supplementary figures and images for: Short-term heavy drinking in a non-human primate model skews monocytes toward a hypo-inflammatory phenotype
Source: Front Immunol. 2025 Jun 23;16:1606092. doi: 10.3389/fimmu.2025.1606092 (PMC12229841; doi:10.3389/fimmu.2025.1606092)

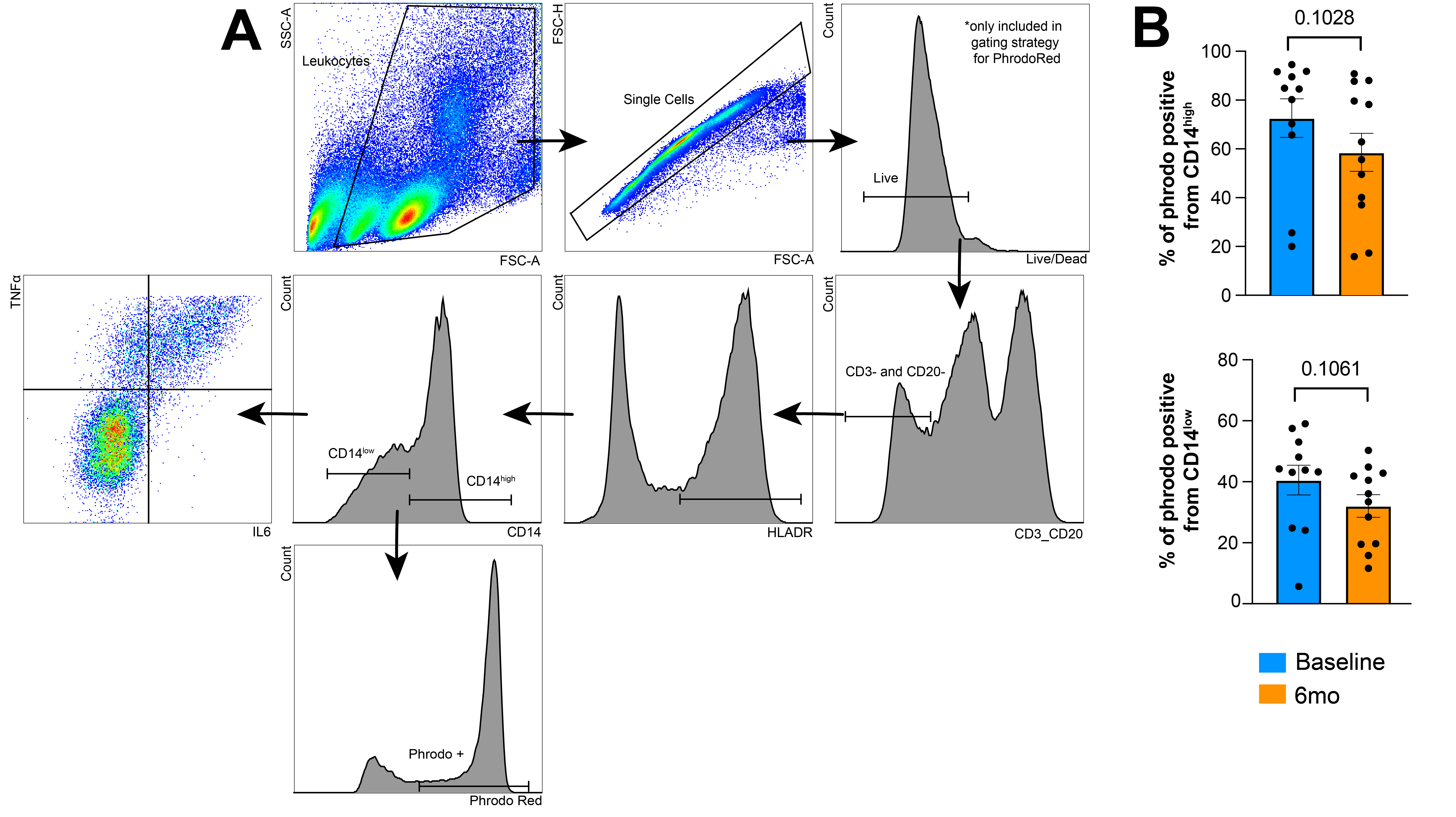

Supplement: Supplementary Figure 1 — CD14high and CD14low monocyte subsets equally contribute to the decrease in phagocytosis after 6mo. (A) Representative gating strategy used to determine the contribution of monocyte subsets to functional response changes. (B) Bar plots representing the percentage of Phrodo Red positive (phrodo+) CD14high and CD14low monocytes. Statistical significance was determined using a linear mixed model, and error bars were defined as ± standard error of the mean (SEM). A p-value of <0.05 was considered significant. [file Image1.jpeg]

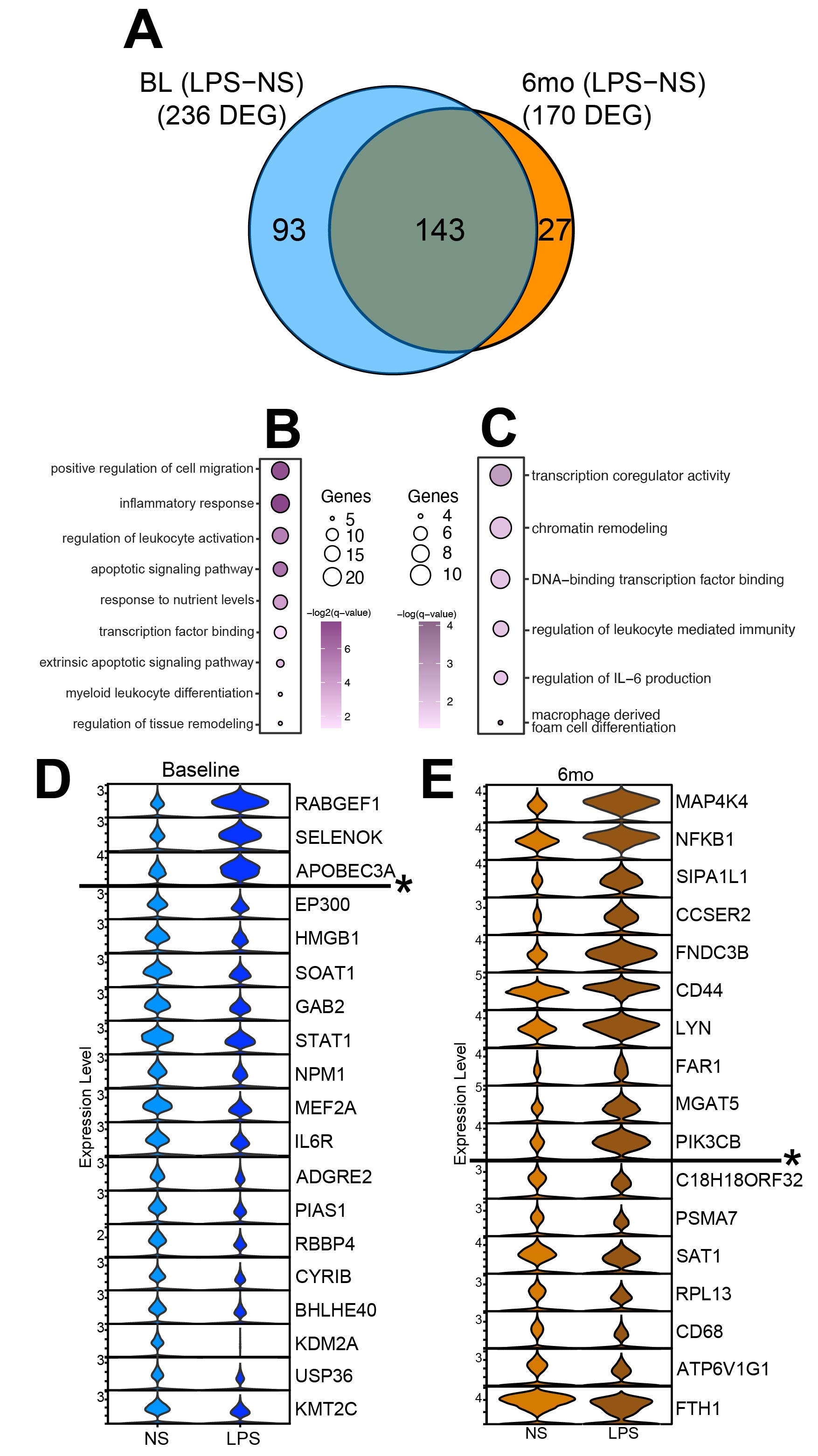

Supplement: Supplementary Figure 2 — Chronic ethanol consumption for 6 months drives differential gene expression in response to LPS. (A) Venn diagram comparing the differentially expressed genes (DEG) in response to LPS before (BL_NS vs. BL_LPS) and after 6mo of alcohol use (6mo_NS vs. 6mo_LPS). (B) Functional enrichments of DEG that were shared between the response to LPS at baseline and after 6mo of drinking and (C) exclusive to baseline response. (D) Select genes specific to LPS response at baseline and (E) after 6mo of heavy drinking. Differentially expressed genes were determined via DESeq2 under default settings in Seurat and enriched using Metascape. Only statistically significant genes (average log(fold-change) cutoff >0.58 or <-0.58; adjusted p-value ≤ 0.05) were included in downstream analysis. [file Image2.jpeg]

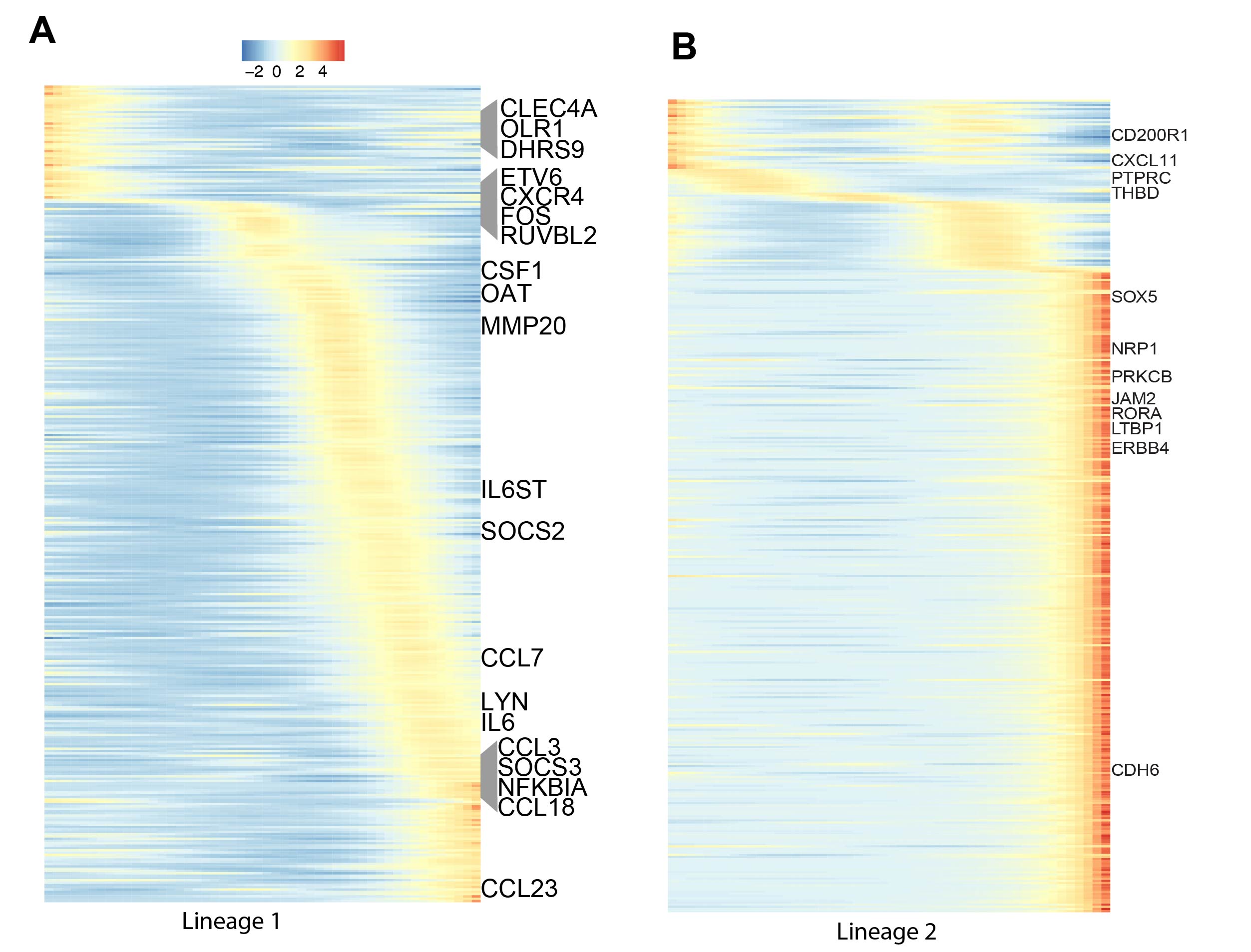

Supplement: Supplementary Figure 3 — LPS stimulation and chronic ethanol consumption induce distinct differentiation trajectories. Heatmap of top 100 genes across pseudotime driving (A) Lineage 1 and (B) Lineage 2. [file Image3.jpeg]

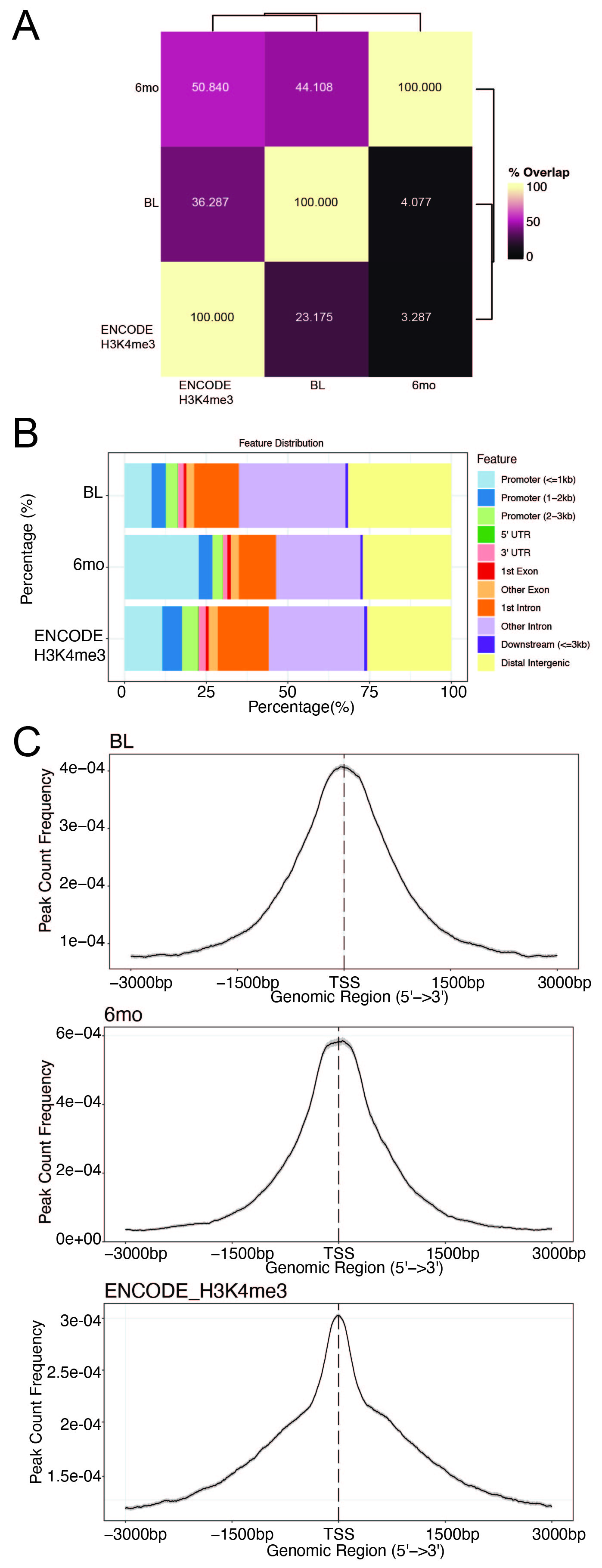

Supplement: Supplementary Figure 4 — Quality control of the CUT&Tag analysis. (A) Correlation of sample peaks to ENCODE monocyte H3k4me3 peak ranges. (B) Genomic distribution of total peaks identified in our samples and the ENCODE monocyte H3k4me3 reference. (C) Peak counts at the transcription start site. [file Image4.jpeg]
